# AI Literacy Among Chinese Medical Students: Cross-Sectional Examination of Individual and Environmental Factors

**DOI:** 10.2196/80604

**Published:** 2026-01-06

**Authors:** Chunqing Li, Sian Hsiang-Te Tsuei, Hongbin Wu

**Affiliations:** 1 Education Management Information Center, Ministry of Education of the People’s Republic of China, Beijing, China; 2 Faculty of Medicine, University of British Columbia, Vancouver, BC, Canada; 3 Institute of Medical Education, Peking University, No 5 Yiheyuan Rd, Beijing, 100191, China, 8601082805597

**Keywords:** medical education, artificial intelligence, AI Literacy, medical student, multidimensional constructs, China

## Abstract

**Background:**

Artificial intelligence (AI) literacy is increasingly essential for medical students. However, without systematic characterization of the relevant components, designing targeted medical education interventions may be challenging.

**Objective:**

This study aimed to systematically describe the levels of and factors associated with multidimensional AI literacy among Chinese medical students.

**Methods:**

A cross-sectional, descriptive analysis was conducted using data from a nationwide survey of Chinese medical students (N=80,335) across 109 medical schools in 2024. AI literacy was assessed with a multidimensional instrument comprising three domains: knowledge, evaluating students’ self-reported proficiency in core areas of medical AI applications; attitude, reflecting their self-perceived views on using AI for teaching and learning; and behavior, capturing the self-perceived usage frequency and application patterns. Multivariate linear regression was applied to examine the associations between individual factors (ie, demographic characteristics, family background, and enrollment motivation) and environmental factors (ie, educational phase, type of education program, and tier of education program) and AI literacy.

**Results:**

Respondents showed moderate to high levels of AI knowledge (mean 76.0, SD 26.9), followed by moderate AI attitude scores (mean 71.6, SD, 24.4). In contrast, AI behavior scores were much lower (mean 32.5, SD, 28.5), indicating little usage of AI tools. Of the individual factors, male students reported higher levels of AI attitude and behavior; both intrinsic and extrinsic motivation were positively associated with all three dimensions; advantaged family background was positively related to AI attitude and behavior, but not knowledge. Among the environmental factors, attending the prestigious Double First-Class universities was positively associated with higher AI usage. Enrollment in long-track medical education programs was associated with higher AI attitude and behavior, while being in the clinical phase was negatively associated with both AI knowledge and behavior. Environmental factors moderated the associations between individual characteristics and AI literacy, potentially attenuating disparities.

**Conclusions:**

Medical students reported moderate to high AI knowledge, moderate AI favorability, and low AI use. Individual characteristics and environmental factors were significantly associated with AI literacy, and environmental factors moderated the associations. The moderate AI literacy overall highlights the need for AI-related medical education, ideally with practical use and nuanced by socioeconomic factors.

## Introduction

Artificial intelligence’s (AI’s) rapid advancement in diagnostic support, treatment decision-making, personalized care, and health system management is quickly reshaping health care service delivery [1-4]. To prepare medical trainees in using these tools [5], medical training programs need to efficiently leverage their limited resources to set up the relevant training approaches and programs to meet the most important needs. However, instead of identifying such gaps, research has mostly focused on applying AI in medical education across tasks, including, for example, admission, research, and evaluation [6-17]. The developments of AI-targeted curricula and competencies have so far advanced without high-quality evidence of trainees’ needs [18-22], highlighting the questionable appropriateness of such proposals.

Among the various AI skills and competencies, clarifying trainees’ AI literacy appears is especially important. AI literacy refers to a set of competencies that enable individuals to critically evaluate AI technologies, communicate and collaborate effectively with AI, and use AI as a tool across various contexts [23]. While various frameworks for AI literacy exist, there is growing consensus that it comprises cognitive knowledge, attitudes, and practical behaviors toward AI tools [5,24-26].

Building on this consensus, we defined AI literacy as a multidimensional construct encompassing knowledge, attitude, and behavior, integrating both classical and contemporary theoretical models. Specifically, this tripartite structure aligns with the ABC model of attitudes in social psychology (Affect–Behavior–Cognition), which conceptualizes human responses as comprising cognitive, affective, and behavioral components [27-29].

Current evidence on AI literacy in medical trainees has been hindered by theoretical and methodological deficiencies. First, the theoretical weakness stems from the lack of recognition that AI literacy has multiple dimensions. Most have focused on AI knowledge [30-33] or attitudes toward AI applications [34-38]. This misses the important dimension of AI behaviors [5,24,25,39,40], which account for the actual usage patterns of AI tools. Second, methodologically, the few studies that captured all three dimensions suffered from capturing only a small number of medical schools and sample size [41,42]. This limits their generalizability. Third, the studies often fail to examine the factors associated with subdimensions of AI literacy, which limits the ability to design targeted interventions within a limited budget. Existing studies have often focused mainly on individual-level variables, overlooking environmental factors associated with the digital divide [41,43-45].

We addressed these gaps by drawing on the comprehensive quantitative database of Chinese medical students, the 2024 China Medical Student Survey (CMSS). We report the distribution of AI literacy across cognitive, attitudinal, and behavioral dimensions among 80,355 Chinese medical students and their associated individual and environmental factors. This large sample size across all three dimensions of AI literacy provides a more meaningful representation of the potential educational needs. We further analyzed the potential factors associated with AI literacy, enabling medical educators to design targeted interventions.

## Methods

### Data Source and Study Sample

Since 2019, China’s National Center for Health Professions Education Development has conducted an annual nationwide survey of medical students in China known as the CMSS. It aims to support medical education development by collecting comprehensive information across the entire educational journey, including students’ demographic backgrounds, preadmission experiences, academic training, and postgraduation plans [46]. The survey takes place between May and July each year. Additional details on the context of medical education in China and the CMSS are provided in Items 1 and 2 in Multimedia Appendix 1. All data analyses adhered to the (STROBE) Strengthening the Reporting of Observational Studies in Epidemiology guidelines for cross-sectional studies.

This study used data from the 2024 CMSS, which included participants from 109 medical schools across 28 provinces in China. The survey achieved a 73.9% response rate (80,355/108,710 surveyed) and captured 109 of 202 medical schools (109/202, 53.9%). The geographical distribution of participating institutions roughly reflected the national pattern. Comparing the dataset and the national distribution, respectively, 49.5% (54/109) and 45.0% (91/202) of the schools were from the eastern region, 28.4% (31/109) and 30.7 % (62/202) from the central region, and 22.0% (24/109) and 24.3% (49/202) from the western region. As for the tiers of institutions, the data included universities’ designation as ‘Double First-Class’ (DFC) institutions. These institutions garner priority investment in China’s bid to establish world-leading educational institutions [47] (see Item 3 in Multimedia Appendix 1 for more details). Further, the proportion of DFC universities was similar in the dataset and nation was (25.7%, 28/109 vs 23.3%, 47/202), respectively.

### Ethical Considerations

The project was approved by the Peking University Institutional Review Board (Beijing, China; approval Number: IRB00001052- 20069). Participation was voluntary, and completion and submission of the questionnaire were considered to constitute informed consent. To protect participant privacy and confidentiality, no personally identifiable information was collected, and all responses were analyzed in an anonymized form. The data were stored securely and were accessible only to the research team. Participants did not receive any financial or other compensation for their participation.

### Measurement of AI Literacy

AI literacy is a multidimensional construct comprising cognitive, attitudinal, and behavioral dimensions [24,25,40]. In this study, we refer to the cognitive dimension as “AI knowledge” to improve clarity and align with common usage in educational research. AI knowledge was assessed through four items evaluating students’ self-perceived proficiency in core areas of medical AI: basic concepts of AI, machine learning tools, multimodal medical data analysis, and ethics issues related to AI. Responses were rated on a three-point scale coded as 1 (low), 2 (medium), and 3 (high) proficiency. Students’ attitude towards AI was measured using two items: students’ self-perceived view of using AI tools in (1) teaching and (2) learning. Responses were rated on a three-point scale, coded as 1 (negative), 2 (neutral), and 3 (positive), respectively. AI behavior refers to actual use of AI tools rather than behavioral intention and was evaluated through self-perceived usage frequency and application patterns. Usage frequency was assessed on a six-point scale (ranging from “never used” to “multiple times daily,” coded one to six, where higher scores indicated more frequent academic use). Usage patterns were measured with two items: extent of using AI for (1) professional knowledge learning and (2) writing tasks (options included “never used,” “generating initial drafts,” “writing specific sections,” “editing and polishing,” “outlining,” and “brainstorming ideas”). The Cronbach α values for cognitive, attitudinal, and behavioral dimension were 0.81, 0.86, and 0.79, respectively, indicating high internal consistency. The Kaiser-Meyer-Olkin values were 0.76, 0.70, and 0.75 for the three dimensions. Additionally, Bartlett’s test of sphericity was significant (*P*<.001), supporting the construct validity of the measurement scales. Further confirmatory factor analysis showed that the measurement instrument loaded well onto the three identified dimensions of AI literacy. Relevant statistics include comparative fit index (CFI)=0.994, Tucker-Lewis index (TLI)=0.991, Root Mean Square Error of Approximation (RMSEA)=0.025, and statistically significant loading on all factors (*P*<.001).

### Factors Associated With AI Literacy

Existing research suggests that both individual and environmental factors associate with access to, and use of digital technologies [45]. For individual factors, we examined demographic characteristics, family background, intellectual ability, and enrollment motivation [47-49]. Demographic characteristics included sex (female or male), ethnicity (Han Chinese or other), only child (yes or no). Family background included hometown (urban or rural), father’s education, mother’s education, having at least one parent as a physician (yes or no), and high-income families (total family income from the previous year>150,000 RMB, approximately US $21,385, yes or no). We used the National College Entrance Examination scores to measure the respondents’ intellectual ability. We assessed enrollment motivation influencing students’ choice of clinical medicine following previous work [47]. Intrinsic motivation was measured by students’ reported interest in medicine, confidence in achieving success in the field, and strong performance in relevant high school subjects. Extrinsic motivation was evaluated through responses about employment prospects, encouragement (or requirements) from significant others, and anticipated convenience in accessing health care resources for themselves and family members.

Regarding environmental factors, we considered students’ affiliation with DFC universities, enrollment in long-track programs (ie, 5+3 or 8 y medical education program instead of 5 y medical education program; yes or no), and the educational phase (preclinical or clinical).

### Statistical Analysis

We used the principal component analysis to aggregate the AI knowledge and AI attitude items, followed by Min-Max normalization to standardize the scores. Based on expert consultation and author consensus, we aggregated the AI behavior index using a weighted arithmetic mean of three key indicators: (1) usage frequency (50% weight), (2) extent of AI integration in professional knowledge learning (30% weight), and (3) AI utilization in writing tasks (20% weight). For robustness check, we also applied equal weights across all three indicators. We then applied the Min-Max normalization method, scaling the data so that the minimum and maximum values correspond to 0 and 100, respectively. Given that students were clustered within schools, we first fitted an unconditional (null) multilevel model to estimate the intraclass correlation coefficient (ICC) and assess the proportion of variance in the standardized AI literacy scores attributable to between-school differences. The ICCs for the three dimensions of AI literacy—knowledge, attitude, and behavior—were 4.0%, 1.0%, and 4.1%, respectively. These results indicate that only a small proportion of the total variance was explained by school-level clustering, suggesting minimal between-school effects. Therefore, we proceeded with multivariate linear regression to examine the associations between individual factors (ie, demographic characteristics, family background, and enrollment motivation) and environmental factors (ie, educational phase, type of education program, and tier of education program) and AI literacy. We conducted subgroup analyses and examined formal interaction terms based on institutional tier. Both classical and school-clustered standard errors were used to calculate 95% CI, and statistical significance was set at a two-sided *P*< 0.05. All analyses were performed using Stata (version 18.0; StataCorp LLC) between December 19, 2024, and March 18, 2025.

## Results

### Participant Characteristics

Of the 80,355 medical students included, most were Han (69,333 students, 86.3%) and female (41,227, 51.3%). A total of 28,885 students (35.9%) reported being the only child; 48,716 (60.6%) were from urban areas; 29,152 (36.3%) reported having at least one parent as a physician. In addition, 13,736 (17.1%) came from high-income families, 36,041 (44.9%) were in the clinical phase of training, and 5823 (7.2%) were enrolled in long-track medical education programs (Table 1).

**Table 1.** Characteristics of participants.

Variables	Participants, N=80,355			
	Non-DFC^a^ (n=67,417)	DFC (n=12,938)	Total (N=80,355)	*P* value
Sex, n (%)				
Female	34563 (51.3)	6664 (51.5)	41227 (51.3)	.62
Male	32854 (48.7)	6274 (48.5)	39128 (48.7)	
Ethnicity, n (%)				
Others	9609 (14.3)	1413 (10.9)	11022 (13.7)	<.001
Han Chinese	57808 (85.7)	11525 (89.1)	69333 (86.3)	
Only child, n (%)				
No	44273 (65.7)	7197 (55.6)	51470 (64.1)	<.001
Yes	23144 (34.3)	5741 (44.4)	28885 (35.9)	
Hometown, n (%)				
Rural	27576 (40.9)	4063 (31.4)	31639 (39.4)	<.001
Urban	39841 (59.1)	8875 (68.6)	48716 (60.6)	
Father’s education, mean (SD)	11.0 (3.7)	12.0 (4.0)	11.1 (3.8)	<.001
Mother’s education, mean (SD)	10.0 (4.1)	11.1 (4.4)	10.2 (4.2)	<.001
Physician parent, n (%)				
No	43146 (64.0)	8057 (62.3)	51203 (63.7)	<.001
Yes	24271 (36.0)	4881 (37.7)	29152 (36.3)	
Family income, n (%)				
Middle and low	57022 (84.6)	9597 (74.2)	66619 (82.9)	<.001
High	10395 (15.4)	3341 (25.8)	13736 (17.1)	
NCEE^b^ score, mean (SD)	546.9 (50.6)	593.7 (54.9)	554.5 (54.1)	<.001
Enrollment motivation, mean (SD)				
Intrinsic motivation	69.9 (18.4)	70.1 (18.6)	69.9 (18.4)	.15
Extrinsic motivation	68.4 (18.6)	67.7 (19.1)	68.3 (18.7)	<.001
Long-track programs^c^, n (%)				
No	64425 (95.6)	10107 (78.1)	74532 (92.8)	<.001
Yes	2992 (4.4)	2831 (21.9)	5823 (7.2)	
Educational phase, n (%)				
Preclinical	37439 (55.5)	6875 (53.1)	44314 (55.1)	<.001
Clinical	29978 (44.5)	6063 (46.9)	36041 (44.9)	

a DFC: Double First-Class universities

b NCEE: National College Entrance Examination

c Long-track programs: the 5+3 medical education program and 8-year medical education program

When stratified by DFC status, students from DFCs came from relatively more advantaged families—with higher household incomes, more parental education, and greater likelihood of urban origin—and also had significantly higher college entrance examination scores (Table 1).

### Distribution of the Three Dimensions of AI Literacy

In our analysis of the three dimensions of AI literacy among Chinese medical students (Table 2), the scores declined from knowledge (mean 76.0, SD 26.9) to attitude (mean 71.6, SD 24.4) and then further to behavior (mean 32.5, SD 28.5). When we stratified by institutional tier, students from DFC universities reported lower AI knowledge (mean 72.6, SD 28.6 vs mean 76.5, SD 26.7; *P*<.001) but higher attitude (mean 72.6, SD 24.6 vs mean 71.4, SD 24.4; *P*<.001), and behavior (mean 35.0, SD 28.4 vs mean 32.1, SD 28.5; *P*<.001).

**Table 2.** Summary results for three dimensions of AI literacy.

Variables	Non-DFC^a^ (n=67,417)	DFC (n=12,938)	Total (N=80,355)	*P* value
AI knowledge, mean (SD)	76.5 (26.7)	72.6 (28.6)	76.0 (26.9)	<.001
AI attitude, mean (SD)	71.4 (24.4)	72.6 (24.6)	71.6 (24.4)	<.001
AI behavior, mean (SD)^b^	32.1 (28.5)	35.0 (28.4)	32.5 (28.5)	<.001
AI behavior, mean (SD)^c^	32.3 (29.5)	35.4 (29.5)	32.8 (29.5)	<.001

a DFC: Double First-Class universities.

b expert -assigned weights.

c equal weights.

**Figure 1.**
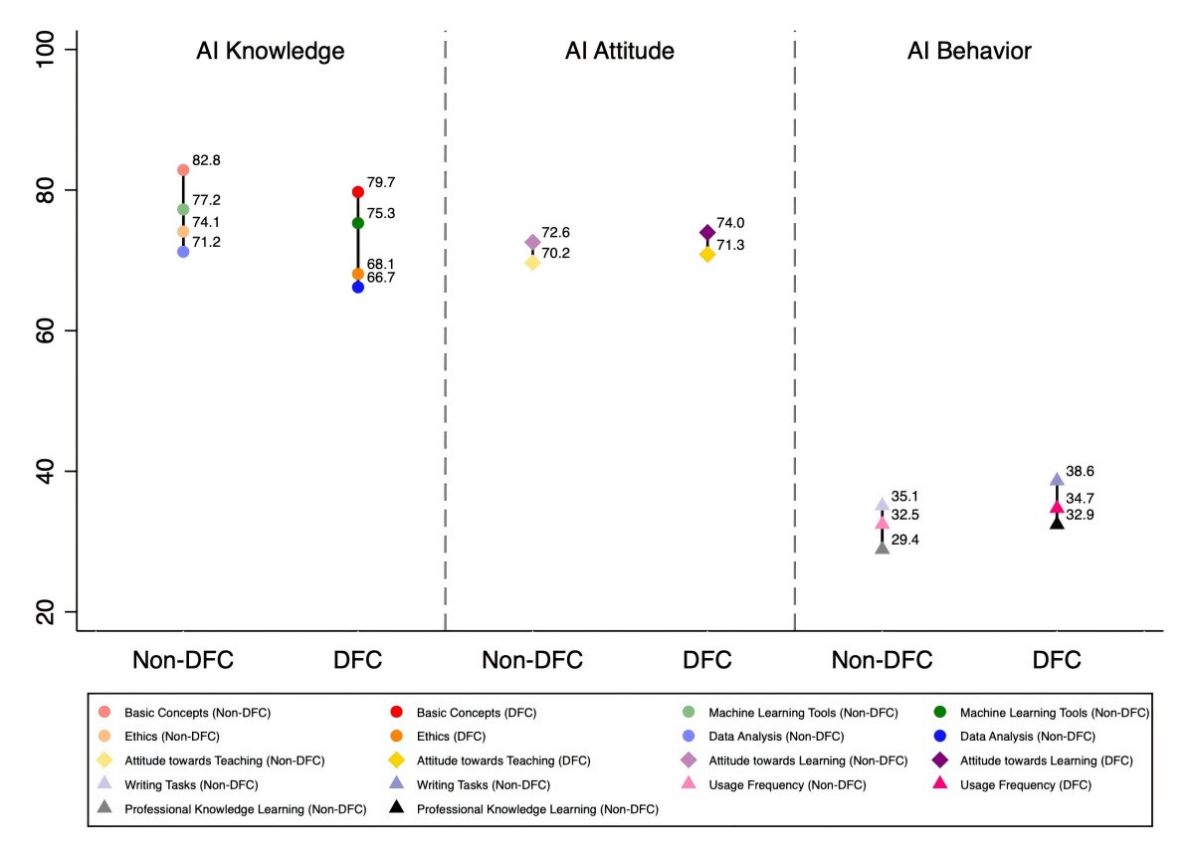
Components of the three dimensions of AI literacy by institutional tier AI: artificial intelligence; DFC: Double First-Class universities. DFC represents advantaged universities, and non-DFC represents less advantaged universities.

Figure 1 further illustrates the item-level patterns within each dimension of AI literacy. The descending trend—from higher scores in knowledge to lower scores in attitude and the lowest in behavior—was consistent across institutional tiers. Notably, students from DFC universities scored higher on AI behavior subcomponents, especially in professional learning and writing tasks. More detailed results on each institution are provided in Figure S1 in Multimedia Appendix 1.

The figure shows the distribution of the subcomponents of the three dimensions of AI literacy by institutional tier. The AI knowledge dimension comprised students’ self-reported proficiency in core areas of medical AI, including (1) basic concepts of AI, (2) machine learning tools, (3) multimodal medical data analysis, and (4) ethics issues related to AI. The AI attitude dimension included students’ view of using AI tools in (1) teaching and (2) learning. The AI behavior dimension was measured by usage frequency and application patterns, including AI use for professional knowledge learning and writing tasks.

### Factors Associated With AI Knowledge

**Figure 2.**
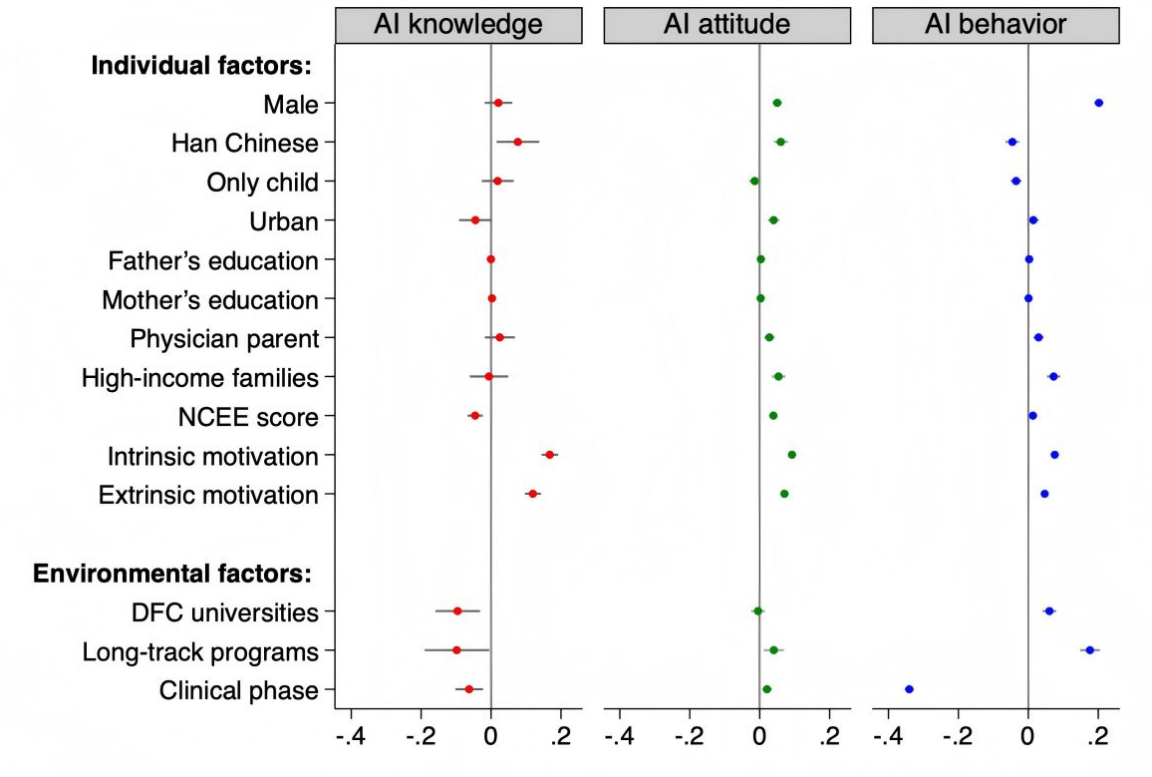
The individual and environmental factors associated with AI literacy in the full sample. AI: artificial intelligence; DFC: Double First-Class; NCEE: National College entrance examination. DFC represents advantaged universities, and non-DFC represents less advantaged universities. Long-track programs include the 5+3 medical education program and 8-year medical education program.

Figure 2 shows the results of regression analyzes of the individual and environmental factors associated with AI literacy (more details including robustness check results are available in Tables S1-S4 in Multimedia Appendix 1). Male students were slightly more likely to report higher AI knowledge (β=0.02, 95% CI –0.02 to 0.06; *P*=.30). Both intrinsic (β=0.17, 95% CI 0.14 to 0.19; *P*<.001) and extrinsic motivation (β=0.12, 95% CI, 0.10 to 0.14; *P*<.001) were positively associated with AI knowledge. Students enrolled in long-track programs reported lower AI knowledge than those in the five-year programs (β=−0.10, 95% CI −0.19 to −0.01; *P*=.04). When stratified by DFC status, these patterns remained largely consistent. However, students from DFC universities reported significantly less AI knowledge (*β*=−0.10, 95% CI −0.16 to −0.03; *P*=.003).

### Factors Associated With AI Attitude

Male students (β=0.05, 95% CI 0.04 to 0.06; *P*<.001) demonstrated significantly more positive attitudes toward AI. Both intrinsic (β=0.09, 95% CI 0.08 to 0.10; *P*<.001) and extrinsic motivation (β=0.07, 95% CI 0.06 to 0.08; *P*<.001) were positively associated with AI attitude. Students from high-income families (β=0.05, 95% CI 0.03 to 0.07; *P*<.001), with at least one physician parent (β=0.03, 95% CI 0.01 to 0.04; *P*<.001), and urban backgrounds (β=0.04, 95% CI 0.02 to 0.06; *P*<.001) showed more positive AI attitude. As for environmental factors, students enrolled in long-track programs (*β*=0.04, 95% CI 0.01 to 0.07; *P*<.006) and in clinical phase of training (*β*=0.02, 95% CI 0.01 to 0.03; *P*<.003) showed significantly more positive attitudes toward AI. These patterns remained largely consistent in subgroup analyses by DFC status; however, among the non-DFC students, the associations of family income and parental medical background on AI attitude were significantly stronger.

### Factors Associated With AI Behavior

Males (β=0.20, 95% CI 0.19 to 0.22; *P*<.001) demonstrated higher levels of AI behavior. Students from high-income families (β=0.07, 95% CI 0.05 to 0.09; *P*<.001) and those with at least one physician parent (β=0.03, 95% CI 0.01 to 0.04; *P*<.001) showed higher AI behavior scores. Both intrinsic (β=0.08, 95% CI 0.07 to 0.08; *P*<.001) and extrinsic motivation (β=0.05, 95% CI 0.04 to 0.05; *P*<.001) were positively associated with AI behavior. Students enrolled in long-track programs (*β*=0.18, 95% CI 0.15 to 0.20; *P*<.001) exhibited higher AI behavior. Again, these patterns were largely held in subgroup analysis by DFC status. In the non-DFC group, however, the effect of family income and parental medical background was again stronger.

**Figure 3.**
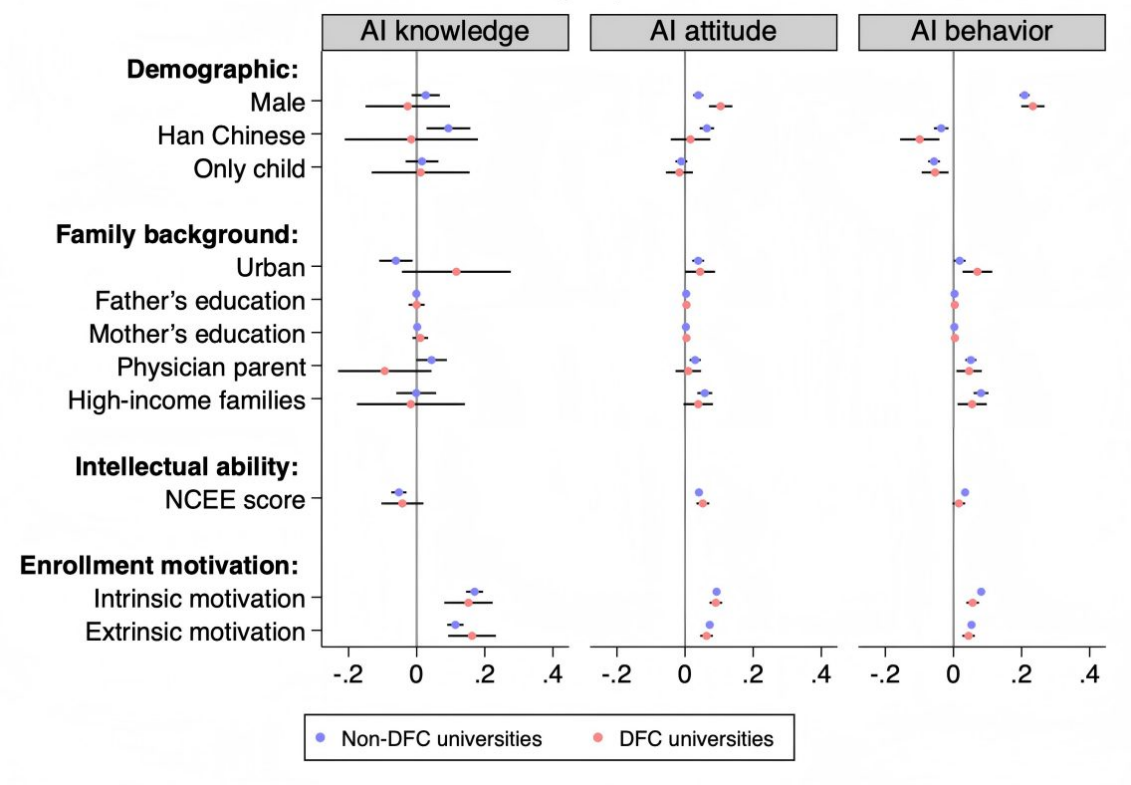
The individual and environmental factors associated with AI literacy. AI: artificial intelligence; DFC: Double First-Class; NCEE: National College entrance examination.

Figure 3 illustrates the associations between individual factors and AI literacy across different institutional environments.

## Discussion

### Principal Findings

Despite the recognized importance of preparing medical students to use AI tools effectively, systematic evidence on the subcomponents of AI literacy and the associated factors is limited. To our knowledge, this is the first large-scale, multicenter, national-level study on the multidimensional constructs of AI literacy and their associated individual and environmental factors among medical students. Three key results emerged. First, medical students reported moderate to high AI knowledge, moderate AI attitude, but only low AI behavior scores. Second, both individual and environmental factors were associated with different dimensions of AI literacy. Third, compared to less favorable environmental conditions, the association between worse AI literacy and family income and parental medical background may be weaker.

This study provides several significant contributions. First, the use of a large, multicenter, and nationally representative sample—covering more than 50% (109/202) of the medical schools in China—expands beyond the small, single-center settings from previous studies. Second, by comprehensively examining the cognitive, attitudinal, and behavioral dimensions of AI literacy, this study provides a more holistic understanding of medical students’ AI literacy profiles, identifying nuanced gaps and interventional needs within AI literacy, particularly around attitude and behaviors. Third, our comprehensive examination of the individual differences and environmental factors associated with AI literacy provides an evidence-based foundation for developing tailored interventions. Understanding these associations enables medical educators to design targeted programs that meet the diverse needs of student populations, potentially reducing disparities in AI literacy and ensuring that all future physicians are adequately prepared to engage with an increasingly AI-integrated health care system.

Our findings consistently revealed that self-perceived AI knowledge scores surpassed attitude and behavior. This suggests that while students developed a foundational understanding of AI and generally perceived AI integration in medicine positively, they still lacked practical experience in its application. Although this defies common logic that deeper knowledge should portend more use [50], this trend aligns with previous international studies of medical staff and trainees who held generally positive attitudes toward AI despite low exposure in formal use [36,51].

The potential barriers underlying this knowledge–use gap are as follows. First, structural barriers—such as limited institutional infrastructure, insufficient curricular integration, and time constraints—may constrain students’ ability to translate AI knowledge into practice [52]. Such challenges are particularly salient in the Chinese healthcare systems, where physician shortages drive each health care provider to carry significant patient load [53]. These contextual limitations can, in turn, dampen students’ autonomous motivation to explore alternative tools [54]. Second, sociocultural norms may also constrain AI use [55]. A recent study showed that nearly half of Chinese medical staff (1320/2705, 48.8%) expressed concerns about the ethical safety, accuracy, interpretability, and fairness of AI systems [56]. Such ethical reservations from staff providers to medical trainees, leading them to perceive AI use as potentially inappropriate, dishonest, unprofessional, or harmful. This may then limit the students’ willingness to use the AI tools despite being knowledgeable of AI tools’ capabilities. suggesting that moral and risk considerations may underlie the limited AI use observed among some medical students. Third, our survey was conducted between May and July 2024, when most mature and user-friendly AI tools were developed abroad. Chinese students may have faced barriers related to access [57] and cultural appropriateness [58].

At an individual level, students’ demographics, family background, and motivation were all associated with AI literacy. The patterns generally aligned with the literature. Demographically, male medical students scored higher in AI attitude and behavior. This is consistent with previous findings [50,59] and the phenomenon of a “technological gender gap” [60]. Male students tended to perceive AI tools as more useful and easier to use [61-64]. Additionally, individuals from advantaged family background (ie, urban residents, those with at least one physician parent, and students from high-income families) perceived AI more favorably and used it more frequently. These advantaged groups hold greater material, cognitive, social, and cultural capital that can facilitate technological adoption [65]. Although technological innovations eventually reach broader populations, disparities in access and adoption persist during the diffusion process, with advantaged groups generally adopting innovations earlier and using them more effectively, thus creating a digital divide between socioeconomic groups [65,66].

Notably, both intrinsic and extrinsic motivation were positively associated with all dimensions of AI literacy, with intrinsic motivation showing particularly strong effects. According to self-determination theory, intrinsic motivation is associated with better performance and enhanced subjective well-being, while extrinsic motivation can also drive performance, even filtering into intrinsic motivation under certain conditions [67-69]. Intrinsic motivation serves as a natural source of AI learning and achievement, fostering greater autonomy, exploration, and reflection that promote deeper AI engagement. In contrast, extrinsically motivated students may preferentially recognize the instrumental value of AI but limit autonomous exploration of AI tools.

Our study highlights the significant role of the environment in shaping AI literacy. Specifically, DFC students demonstrated higher AI behavior scores. The DFC Project represents China’s willingness to invest financial and human resources in these institutions to develop world-class universities and disciplines [47,70]. The stronger student–faculty and peer interactions and higher-quality student efforts may promote deeper engagement with AI [71]. The seemingly paradoxical finding that DFC students reported lower AI knowledge but higher AI behavior may stem from the Dunning–Kruger effect [72,73]. More competent individuals tend to recognize the complexity of a domain and their own knowledge limitations, leading to more cautious self-assessments, while those less competent may overestimate their understanding. DFC students, exposed to higher levels of academic research, may be more aware of the challenges inherent in AI, whereas non-DFC students, with relatively limited exposure, may exhibit overconfidence in their basic knowledge. Similar cognitive bias patterns have been observed among health professions students; for example, low-performing medical students have been shown to substantially overestimate their academic performance relative to peers [74,75].

Importantly, in these DFC supportive environments, the students’ family income and parental medical background showed less pronounced associations with AI attitude and behavior, suggesting that the supportive learning environment may have mitigated the detrimental relationship between students’ background and AI literacy [48].

In addition, the students’ program also appears to matter. Students in the long-track program demonstrated significantly higher AI attitude and behavior scores. They typically represent China’s elite medical students, attending research-oriented universities that provide enhanced research training opportunities and funding [76], potentially including familiarization with AI technologies that led to more positive perceptions and use of AI tools. The students’ phase of training also appeared to be associated with their AI literacy. Students undergoing clinical training reported higher attitude scores but lower knowledge and behavior scores. Perhaps the complexity of clinical practice and the pressure of professional development precipitated cognitive overload and a preferential reliance on traditional tools [77]. The technical limitations and evolving normative and legal thresholds around AI technologies may further exacerbate technology avoidance [78-82].

Tying together the findings, we provide five sets of recommendations to help medical schools maneuver through the current lack of structured AI education and training [83]. First, carefully integrating AI-related content into the curriculum to generate practical AI exposure may help bridge the knowledge-use gap. This should be mindful of the potentially detrimental association with excessive cognitive offloading [84] while ensuring a minimal knowledge and skills threshold [85]. As regulations and ethical frameworks surrounding AI use in health care continue to evolve to meet the technical, ethical, and legal challenges [83,86,87], developing interdisciplinary training on practical risks, benefits, and guidelines on AI usage may help establish norms around AI usage. Second, developing and implementing scalable AI education frameworks that enable targeted training based on learners’ backgrounds can potentially reduce inequities in AI literacy driven by individual and environmental differences while maximizing educational impact.

The next three recommendations focus specifically on digital divide, and we draw on Han and Kumwenda’s framework for mitigating digital divide. The framework considers first-order barriers relate to infrastructure and access; second-order barriers include motivation and engagement; and third-order barriers reflect skill and training disparities [88].

First, medical educators and policy makers should prioritize investments in digital infrastructure to reduce disparities in technological access across institutional and regional contexts [89]. Targeted funding and digital resource allocation can ensure that students in under-resourced settings are not excluded from AI-driven learning environments.

Second, improving motivation and engagement may incorporate experiential learning opportunities [87,90,91]. Providing all students—particularly those from disadvantaged backgrounds—with the chance to work directly with AI tools and integrate them into their coursework can foster greater motivation, engagement, and confidence in using AI technologies.

Third, structured and scalable AI education frameworks that offer tiered and targeted training that adapts to learners’ backgrounds. Such approaches would focus on ensuring that disadvantaged students can develop foundational AI literacy before advancing to higher-order skills. Moreover, the development of multilingual and culturally adaptive AI models [92] can further ensure inclusivity and contextual relevance in AI-enhanced medical education.

### Limitations

First, the reliance on self-reported data potentially exposes the study to social desirability bias. It is necessary to develop objective instruments to measure medical students’ AI literacy. Such a tool can help evaluate AI proficiency based on observable AI use. Performance- and scenario-based assessments might be potential avenues to operationalize such evaluation. Second, given the rapid development of AI technologies, the AI literacy of medical students is likely to evolve rapidly. To capture these dynamic shifts of AI literacy, we are actively exploring another round of survey. Third, this study employed a cross-sectional design, which precludes causal inference. Although most individual and environmental factors were determined before the measurement of AI literacy, reverse causality cannot be fully excluded, and unmeasured confounders may have influenced both the predictors and outcomes, potentially biasing the observed associations. Fourth, even though the unique premedical training structure limits the generalizability of the findings, the methodological framework and multidimensional conceptualization of AI literacy (knowledge, attitude, and behavior) may still provide a useful reference for future comparative studies or adaptations in other educational systems.

### Conclusions

This cross-sectional study revealed that medical students exhibited the highest performance in AI knowledge, followed by attitude, and then behavior. Both individual characteristics and environmental factors were significantly associated with AI literacy, and environmental factors moderated individual variations in AI literacy. Integrating practical AI-related training into medical curricula through interdisciplinary collaboration, coupled with targeted interventions for students according to their backgrounds, may help prepare future physicians to effectively engage with AI technologies in medical practice.

## Supplementary material

10.2196/80604 Multimedia Appendix 1

## Abbreviations

AI: artificial intelligence
CMSS: China Medical Student Survey
DFC: Double First-Class
ICC: intraclass correlation coefficient

## References

[1] Elemento O, Khozin S, Sternberg CN (2025). The use of artificial intelligence for cancer therapeutic decision-making. NEJM AI.

[2] Sarkar U, Bates DW (2024). Using artificial intelligence to improve primary care for patients and clinicians. JAMA Intern Med.

[3] Haug CJ, Drazen JM (2023). Artificial intelligence and machine learning in clinical medicine, 2023. N Engl J Med.

[4] Unlu O, Varugheese M, Shin J (2025). Manual vs AI-assisted prescreening for trial eligibility using large language models-a randomized clinical trial. JAMA.

[5] Boscardin CK, Gin B, Golde PB, Hauer KE (2024). ChatGPT and generative artificial intelligence for medical education: potential impact and opportunity. Acad Med.

[6] Preiksaitis C, Rose C (2023). Opportunities, challenges, and future directions of generative artificial intelligence in medical education: scoping review. JMIR Med Educ.

[7] Gordon M, Daniel M, Ajiboye A (2024). A scoping review of artificial intelligence in medical education: BEME Guide No. 84. Med Teach.

[8] Burk-Rafel J, Reinstein I, Feng J (2021). Development and validation of a machine learning-based decision support tool for residency applicant screening and review. Acad Med.

[9] Baron T, Grossman RI, Abramson SB (2020). Signatures of medical student applicants and academic success. PLoS One.

[10] Kind T, Olvet DM, Farina G (2021). Reading and study habits of medical students on clerkships and performance outcomes: a multi-institutional study. Med Sci Educ.

[11] Cutrer WB, Spickard WA, Triola MM (2021). Exploiting the power of information in medical education. Med Teach.

[12] Roberts LW (2024). Addressing the novel implications of generative ai for academic publishing, education, and research. Acad Med.

[13] Stokel-Walker C (2023). ChatGPT listed as author on research papers: many scientists disapprove. Nature New Biol.

[14] Flanagin A, Bibbins-Domingo K, Berkwits M, Christiansen SL (2023). Nonhuman “Authors” and implications for the integrity of scientific publication and medical knowledge. JAMA.

[15] Tolsgaard MG, Boscardin CK, Park YS, Cuddy MM, Sebok-Syer SS (2020). The role of data science and machine learning in Health Professions Education: practical applications, theoretical contributions, and epistemic beliefs. Adv Health Sci Educ Theory Pract.

[16] Gin BC, Ten Cate O, O’Sullivan PS, Hauer KE, Boscardin C (2022). Exploring how feedback reflects entrustment decisions using artificial intelligence. Med Educ.

[17] Schaye V, Guzman B, Burk-Rafel J (2022). Development and validation of a machine learning model for automated assessment of resident clinical reasoning documentation. J Gen Intern Med.

[18] Triola MM, Rodman A (2025). Integrating generative artificial intelligence into medical education: curriculum, policy, and governance strategies. Acad Med.

[19] Russell RG, Lovett Novak L, Patel M (2023). Competencies for the use of artificial intelligence–based tools by health care professionals. Acad Med.

[20] Ong QC, Ang CS, Lai NM, Neves AL, Car J (2025). Dearth of digital health education: the need for an accelerated medical curriculum reform in Malaysia. Lancet Reg Health West Pac.

[21] Succi MD, Chang BS, Rao AS (2025). Building the AI-enabled medical school of the future. JAMA.

[22] Lee J, Wu AS, Li D, Kulasegaram K (Mahan (2021). Artificial intelligence in undergraduate medical education: a scoping review. Acad Med.

[23] Long D, Magerko B (2020). What is AI literacy? Competencies and design considerations. CHI ’20: Proceedings of the 2020 CHI Conference on Human Factors in Computing Systems.

[24] Ng DTK, Wu W, Leung JKL, Chiu TKF, Chu SKW (2024). Design and validation of the ai literacy questionnaire: the affective, behavioural, cognitive and ethical approach. Brit J Educational Tech.

[25] Ng DTK, Leung JKL, Chu SKW, Qiao MS (2021). Conceptualizing AI literacy: an exploratory review. Computers and Education: Artificial Intelligence.

[26] Aktoprak A, Hursen C (2022). A bibliometric and content analysis of critical thinking in primary education. Think Skills Creat.

[27] Gruber T, Bazhydai M, Sievers C, Clément F, Dukes D (2022). The ABC of social learning: affect, behavior, and cognition. Psychol Rev.

[28] Boyle MJ, Williams B, Brown T (2010). Attitudes of undergraduate health science students towards patients with intellectual disability, substance abuse, and acute mental illness: a cross-sectional study. BMC Med Educ.

[29] Van Harreveld F (2015). Adv Exp Soc Psychol.

[30] Patino GA, Amiel JM, Brown M, Lypson ML, Chan TM (2024). The promise and perils of artificial intelligence in health professions education practice and scholarship. Acad Med.

[31] Barreiro-Ares A, Morales-Santiago A, Sendra-Portero F, Souto-Bayarri M (2023). Impact of the rise of artificial intelligence in radiology: What do students think?. Int J Environ Res Public Health.

[32] Stewart J, Lu J, Gahungu N (2023). Western Australian medical students’ attitudes towards artificial intelligence in healthcare. PLoS One.

[33] Wood EA, Ange BL, Miller DD (2021). Are we ready to integrate artificial intelligence literacy into medical school curriculum: students and faculty survey. J Med Educ Curric Dev.

[34] Ejaz H, McGrath H, Wong BL, Guise A, Vercauteren T, Shapey J (2022). Artificial intelligence and medical education: a global mixed-methods study of medical students’ perspectives. Digit Health.

[35] Doumat G, Daher D, Ghanem NN, Khater B (2022). Knowledge and attitudes of medical students in Lebanon toward artificial intelligence: A national survey study. Front Artif Intell.

[36] Sit C, Srinivasan R, Amlani A (2020). Attitudes and perceptions of UK medical students towards artificial intelligence and radiology: a multicentre survey. Insights Imaging.

[37] Mehta N, Harish V, Bilimoria K (2021). Knowledge of and attitudes on artificial intelligence in healthcare: a provincial survey study of medical students. Medical Education.

[38] Moldt JA, Festl-Wietek T, Madany Mamlouk A, Nieselt K, Fuhl W, Herrmann-Werner A (2023). Chatbots for future docs: exploring medical students’ attitudes and knowledge towards artificial intelligence and medical chatbots. Med Educ Online.

[39] Teng M, Singla R, Yau O (2022). Health care students’ perspectives on artificial intelligence: countrywide survey in Canada. JMIR Med Educ.

[40] Wang B, Rau PLP, Yuan T (2023). Measuring user competence in using artificial intelligence: validity and reliability of artificial intelligence literacy scale. Behaviour & Information Technology.

[41] Laupichler MC, Aster A, Meyerheim M, Raupach T, Mergen M (2024). Medical students’ AI literacy and attitudes towards AI: a cross-sectional two-center study using pre-validated assessment instruments. BMC Med Educ.

[42] Swed S, Alibrahim H, Elkalagi NKH (2022). Knowledge, attitude, and practice of artificial intelligence among doctors and medical students in Syria: A cross-sectional online survey. Front Artif Intell.

[43] Holstein K, Doroudi S (2021). Equity and artificial intelligence in education: will “AIED” amplify or alleviate inequities in education?. arXiv.

[44] van Deursen AJ, van Dijk JA (2014). The digital divide shifts to differences in usage. New Media & Society.

[45] van Dijk J, Rössler P, Hoffner CA, Zoonen L (2017). The International Encyclopedia of Media Effects.

[46] Ma X, Shen Z, Xiao R, Wu H (2024). Perceived mistreatment and professional identity of medical students in China. JAMA Netw Open.

[47] Wu H, Li S, Zheng J, Guo J (2020). Medical students’ motivation and academic performance: the mediating roles of self-efficacy and learning engagement. Med Educ Online.

[48] Loh RSM, Kraaykamp G, van Hek M (2025). Plugging in at school: do schools nurture digital skills and narrow digital skills inequality?. Computers & Education.

[49] Hargittai E (2010). Digital na(t)ives? Variation in internet skills and uses among members of the “Net Generation”. Sociol Inq.

[50] Pinto dos Santos D, Giese D, Brodehl S (2019). Medical students’ attitude towards artificial intelligence: a multicentre survey. Eur Radiol.

[51] Sarwar S, Dent A, Faust K (2019). Physician perspectives on integration of artificial intelligence into diagnostic pathology. NPJ Digit Med.

[52] Kennedy T, Regehr G, Rosenfield J, Roberts SW, Lingard L (2004). Exploring the gap between knowledge and behavior: a qualitative study of clinician action following an educational intervention. Acad Med.

[53] Wang X, Sanders HM, Liu Y (2023). ChatGPT: promise and challenges for deployment in low- and middle-income countries. The Lancet Regional Health - Western Pacific.

[54] Ryan RM, Deci EL Encyclopedia of Quality of Life and Well-Being Research.

[55] Kahlke RM, McConnell MM, Wisener KM, Eva KW (2020). The disconnect between knowing and doing in health professions education and practice. Adv in Health Sci Educ.

[56] Dai Q, Li M, Yang M (2025). Attitudes, perceptions, and factors influencing the adoption of AI in health care among medical staff: nationwide cross-sectional survey study. J Med Internet Res.

[57] Meaningful connectivity: a new target to raise the bar for internet access. Alliance for Affordable Internet.

[58] Cao Y, Zhou L, Lee S, Cabello L, Chen M, Hershcovich D (2023). Assessing cross-cultural alignment between chatgpt and human societies: an empirical study.

[59] Akca Sumengen A, Ozcevik Subasi D, Cakir GN (2025). Nursing students’ attitudes and literacy toward artificial intelligence: a cross-sectional study. Teaching and Learning in Nursing.

[60] Canada K, Brusca F (1991). The technological gender gap: evidence and recommendations for educators and computer-based instruction designers. ETR&D.

[61] Cai Z, Fan X, Du J (2017). Gender and attitudes toward technology use: a meta-analysis. Comput Educ.

[62] Davis FD (1989). Perceived usefulness, perceived ease of use, and user acceptance of information technology. MIS Q.

[63] Ong CS, Lai JY (2006). Gender differences in perceptions and relationships among dominants of e-learning acceptance. Comput Human Behav.

[64] Ackerman PL, Wolman SD (2007). Determinants and validity of self-estimates of abilities and self-concept measures. J Exp Psychol Appl.

[65] van Dijk J (2020).

[66] Norris P (2003). Digital divide: civic engagement, information poverty, and the internet worldwide. Canadian Journal of Communication.

[67] Ryan RM, Deci EL (2000). Intrinsic and extrinsic motivations: classic definitions and new directions. Contemp Educ Psychol.

[68] Deci EL, Ryan RM (1985). Intrinsic Motivation and Self-Determination in Human Behavior.

[69] Ryan RM, Stiller J (1991). Advances in Motivation and Achievement.

[70] Qi W (2017). Comment: Programmed to fulfill global ambitions. Nature New Biol.

[71] Pascarella ET, Terenzini PT (2005). How College Affects Students: A Third Decade of Research.

[72] Kruger J, Dunning D (1999). Unskilled and unaware of it: how difficulties in recognizing one’s own incompetence lead to inflated self-assessments. J Pers Soc Psychol.

[73] Dunning D (2011). Adv Exp Soc Psychol.

[74] Edwards RK, Kellner KR, Sistrom CL, Magyari EJ (2003). Medical student self-assessment of performance on an obstetrics and gynecology clerkship. Am J Obstet Gynecol.

[75] Austin Z, Gregory PAM, Galli M (2008). “I just don’t know what I’m supposed to know”: Evaluating self-assessment skills of international pharmacy graduates in Canada. Research in Social and Administrative Pharmacy.

[76] Zhang G, Wu H, Xie A, Cheng H (2022). The association between medical student research engagement with learning outcomes. Med Educ Online.

[77] Sweller J (1988). Cognitive load during problem solving: effects on learning. Cogn Sci.

[78] Bernstein MH, Sheppard B, Bruno MA, Lay PS, Baird GL (2025). Randomized study of the impact of AI on perceived legal liability for radiologists. NEJM AI.

[79] Tsuei SHT (2025). How are Canadians regulating artificial intelligence for healthcare? A brief analysis of the current legal directions, challenges and deficiencies. Healthc Pap.

[80] Blumenthal D, Patel B (2024). The regulation of clinical artificial intelligence. NEJM AI.

[81] Topol EJ (2019). High-performance medicine: the convergence of human and artificial intelligence. Nat Med.

[82] Reddy S (2022). Explainability and artificial intelligence in medicine. Lancet Digit Health.

[83] Car J, Ong QC, Erlikh Fox T (2025). The digital health competencies in medical education framework: an international consensus statement based on a Delphi study. JAMA Netw Open.

[84] Kosmyna N, Hauptmann E, Yuan YT (2025). Your brain on ChatGPT: accumulation of cognitive debt when using an AI assistant for essay writing task. arXiv.

[85] Gin BC, LaForge K, Burk-Rafel J, Boscardin CK (2025). Macy Foundation Innovation Report Part II: from hype to reality: innovators’ visions for navigating AI integration challenges in medical education. Acad Med.

[86] Zhai X, Chu X, Chai CS (2021). A review of artificial intelligence (AI) in education from 2010 to 2020. Complexity.

[87] McCoy LG, Nagaraj S, Morgado F, Harish V, Das S, Celi LA (2020). What do medical students actually need to know about artificial intelligence?. NPJ Digit Med.

[88] Han SP, Kumwenda B (2025). Bridging the digital divide: promoting equal access to online learning for health professions in an unequal world. Med Educ.

[89] Zainal H, Xiao Hui X, Thumboo J, Kok Yong F (2025). Organizational leaders’ views on digital health competencies in medical education: qualitative semistructured interview study. JMIR Med Educ.

[90] Paranjape K, Schinkel M, Nannan Panday R, Car J, Nanayakkara P (2019). Introducing artificial intelligence training in medical education. JMIR Med Educ.

[91] Kolachalama VB, Garg PS (2018). Machine learning and medical education. NPJ Digit Med.

[92] Fitas R (2025). Inclusive education with AI: supporting special needs and tackling language barriers. SSRN.

