# Supplementary material for: AI Literacy Among Chinese Medical Students: Cross-Sectional Examination of Individual and Environmental Factors
Source: JMIR Med Educ. 2026 Jan 6;12:e80604. doi: 10.2196/80604 (PMC12772583; doi:10.2196/80604)
Supplement: Multimedia Appendix 1 [file mededu-v12-e80604-s001.doc]

# **Item 1. Context: Medical education in China**

In China, medical education typically begins at the undergraduate level. Students are admitted to medical schools based on their scores in the National College Entrance Examination (NCEE). The system offers three primary training pathways: the 5-year bachelor’s program, the 5+3 master’s program, and the 8-year Doctor of Medicine (MD) program. Among these, the 5-year track remains the most prevalent and represents the standard route for medical training. The 8-year MD program is structured to develop physician-scientists, whereas the 5+3 program is expected to become the central model for future reforms. Students enrolled in the 5-year program complete a broad curriculum covering general education, basic medical sciences, clinical training, and clerkship. In contrast, those in the 8-year or 5+3 tracks transition automatically into postgraduate education or standardized residency training after the fifth year, without requiring separate applications. Students in the 5-year track, however, must independently apply for further training opportunities.^1^

The undergraduate medical education system operates as a relatively closed pipeline, where admission from high school into medical school represents the main entry point for those seeking to become physicians. Moreover, a bachelor's degree in medicine is a prerequisite for taking the National Medical Licensing Examination (NMLE).^2^ Only those who pass the exam receive a physician qualification certificate, which formally certifies their entry into the medical profession. Because medical education is the only pathway to becoming a physician, the number of students accepted into medical programs effectively determines the maximum potential supply of licensed physicians in the country.^3^

# **Item 2. The China Medical Student Survey (CMSS)**

The China Medical Student Survey (CMSS) is a nationwide initiative led by the National Center for Health Professions Education Development (NCHPED), in partnership with the Association for Health Professions Education Research in China (AHPERC). Designed to evaluate the quality of undergraduate medical education from the student perspective, the CMSS collects comprehensive data on students’ backgrounds, academic experiences, and educational outcomes. The survey spans the full training continuum—from pre-admission profiles to curriculum design, institutional support, teaching effectiveness, and clinical training environments—offering an in-depth view of the factors shaping educational quality across medical schools and their affiliated hospitals.^4^

In March of every year, NCHPED invites medical schools to participate and requires them to designate trained project managers responsible for survey coordination and data quality. Managers access a secure online platform (<https://medudata.meduc.cn/>) to oversee administration. Students typically complete the survey between May and June of each year. Participation is voluntary and anonymous; students provide informed consent before submission and are assured that their responses remain confidential and will not influence academic evaluations. They may withdraw at any point without penalty. After the survey closes, NCHPED shares detailed institutional reports summarizing students’ learning experiences and developmental outcomes. These reports support evidence-based educational reform and promote continued engagement in the CMSS, fostering quality enhancement across China’s medical education system.^5^

# **Item 3. The Double First-Class Project**

The Double First-Class Project is a national strategic initiative aimed at strengthening the overall capacity and global competitiveness of China’s higher education system, as well as advancing the country’s development into a world leader in education and human capital. Launched by the government in 2015, the initiative was formalized in early 2017 when the Ministry of Education, the Ministry of Finance, and the National Development and Reform Commission jointly issued the Implementation Plan for Coordinated Development of First-Class Universities and First-Class Disciplines.

Structured in five-year cycles, the project supports both elite universities and approximately 100 selected disciplines deemed to have high development potential. This reflects a strategic emphasis on differentiated development within China’s higher education landscape, encouraging institutions to leverage their unique strengths. Funding is prioritized for three categories: academic departments approaching global excellence, disciplines aligned with national socioeconomic priorities, and emerging interdisciplinary platforms.

The project also promotes a diversified funding structure. Central universities are primarily financed by national funds, while local universities receive core funding from provincial governments. Provincial governments are further encouraged to mobilize resources from communities and industry partners to establish sustainable, long-term funding mechanisms. Resource allocation is performance-based and subject to dynamic adjustments through mid-term reviews and end-of-cycle evaluations. Institutions that fail to meet benchmarks may face funding reductions or, in severe cases, removal from the project list.^6-8^

# **Figure S1. The AI Literacy Across Institutions**


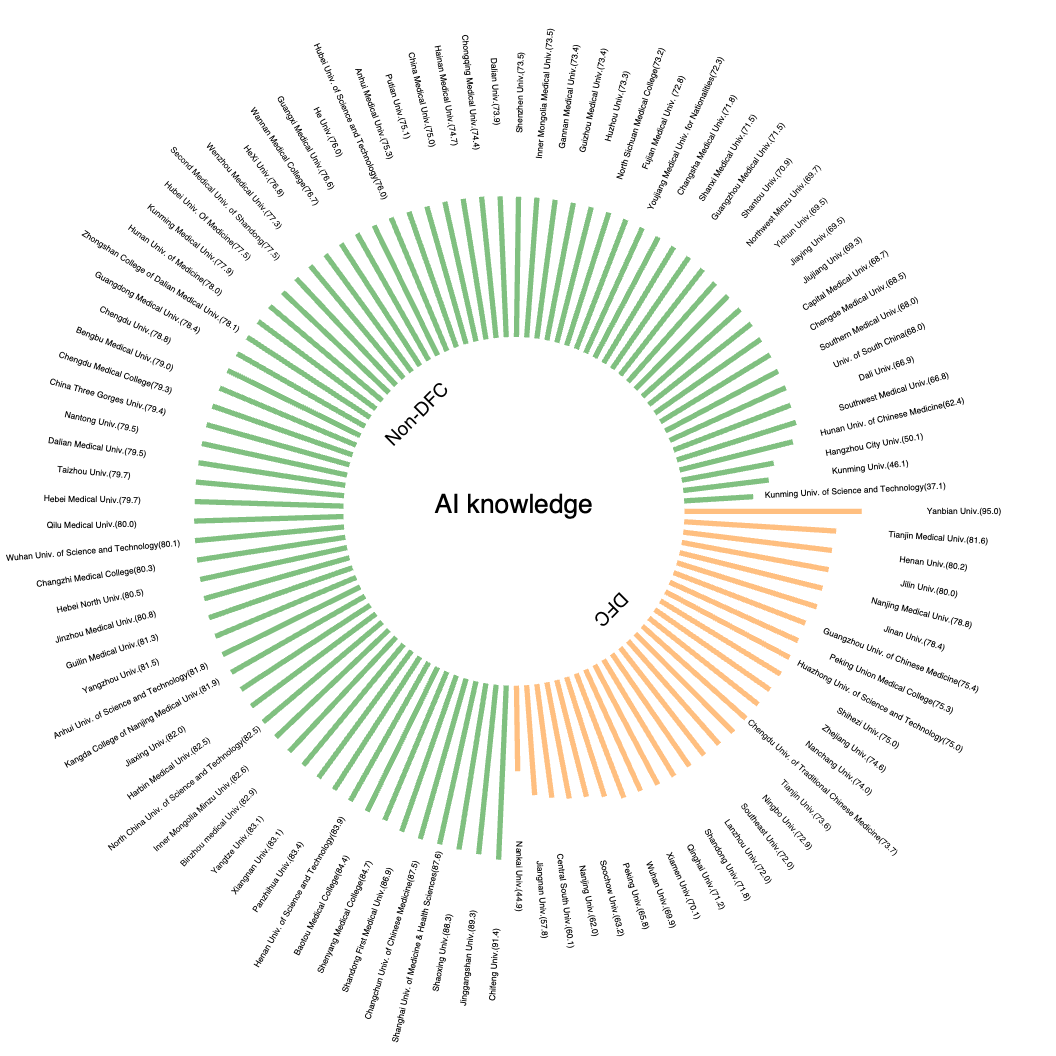

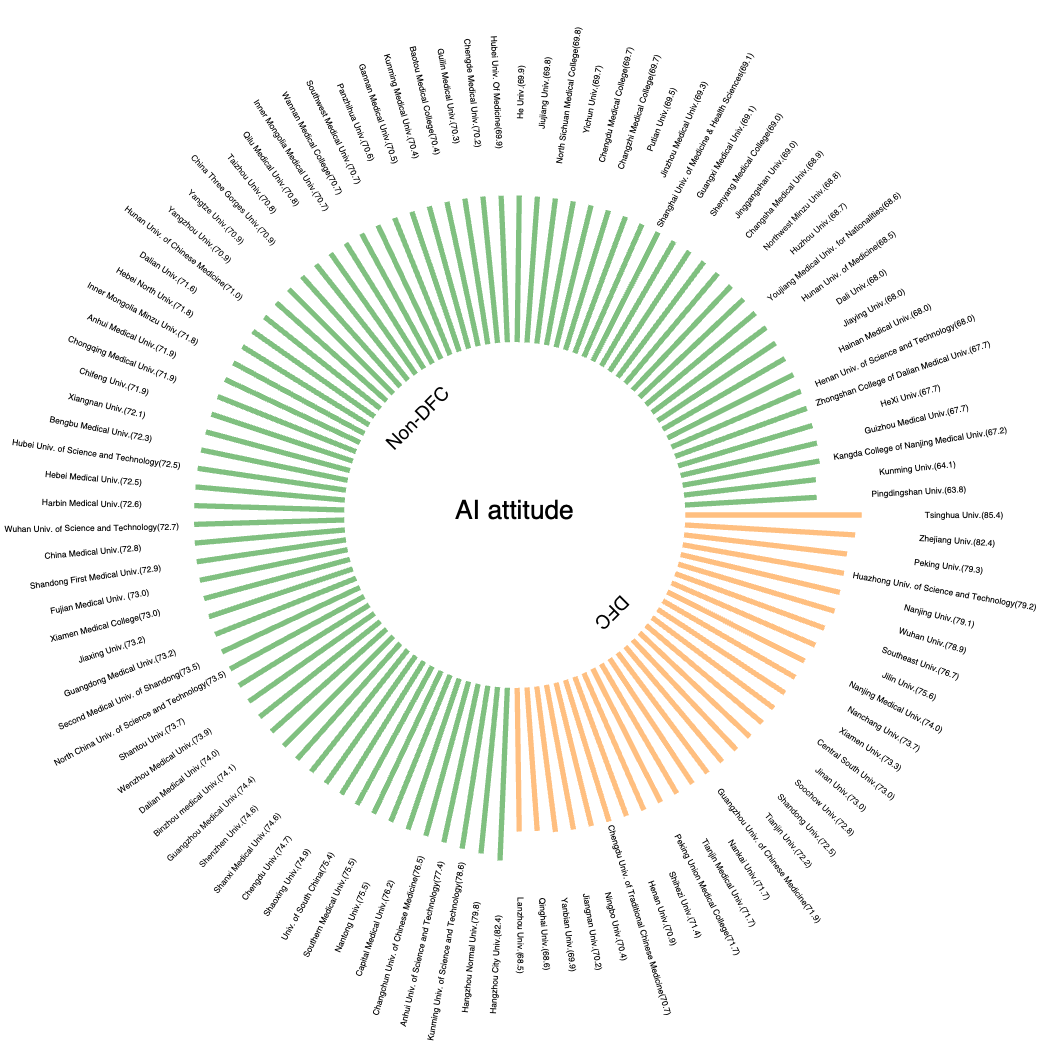

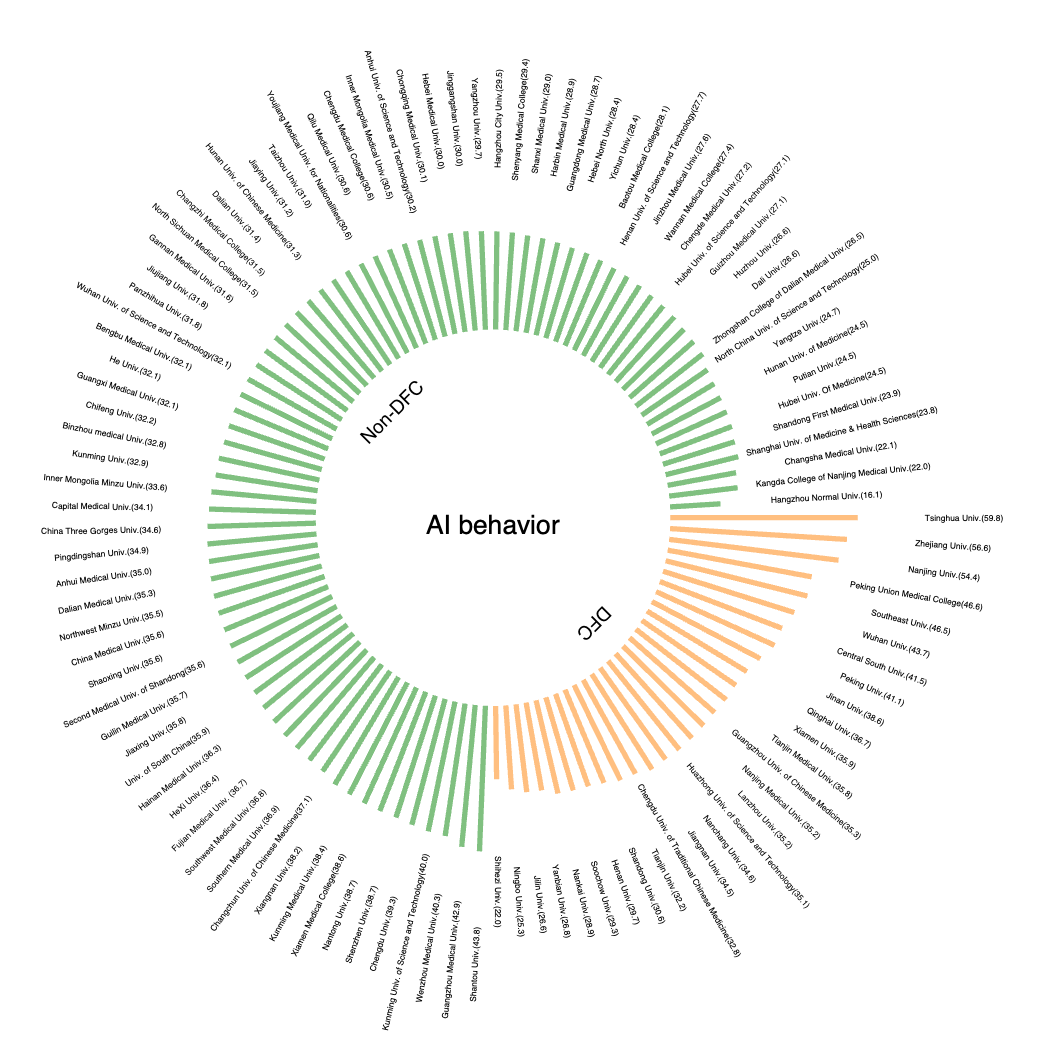


Abbreviations: DFC = Double First-Class universities. DFC represents advantaged universities, and non-DFC represents less advantaged universities.

# Table S1. The Individual and Environmental Factors Associated with AI Literacy

|  | **AI knowledge** | | | | **AI attitude** | | | | **AI behavior** | | | |
| --- | --- | --- | --- | --- | --- | --- | --- | --- | --- | --- | --- | --- |
|  | **Classical SE** | | **Clustered SE ^a^** | | **Classical SE** | | **Clustered SE ^a^** | | **Classical SE** | | **Clustered SE ^a^** | |
|  | **β (95% CI)** | ***p*** | **β (95% CI)** | ***p*** | **β (95% CI)** | ***p*** | **β (95% CI)** | ***p*** | **β (95% CI)** | ***p*** | **β (95% CI)** | ***p*** |
| **Individual factors** |  |  |  |  |  |  |  |  |  |  |  |  |
| Male | 0.02 (-0.02 to 0.06) | .30 | 0.02 (-0.03 to 0.08) | .44 | 0.05 (0.04 to 0.06) | < .001 | 0.05 (0.03 to 0.07) | < .001 | 0.20 (0.19 to 0.22) | < .001 | 0.20 (0.18 to 0.22) | < .001 |
| Han Chinese | 0.08 (0.02 to 0.14) | .01 | 0.08 (0.01 to 0.14) | .02 | 0.06 (0.04 to 0.08) | < .001 | 0.06 (0.03 to 0.09) | < .001 | -0.05 (-0.07 to -0.03) | < .001 | -0.05 (-0.10 to 0.01) | .08 |
| Only child | 0.02 (-0.03 to 0.06) | .42 | 0.02 (-0.03 to 0.07) | .42 | -0.01 (-0.03 to 0.00) | .09 | -0.01 (-0.04 to 0.01) | .19 | -0.04 (-0.05 to -0.02) | < .001 | -0.04 (-0.06 to -0.01) | .01 |
| Urban | -0.04 (-0.09 to 0.00) | .06 | -0.04 (-0.09 to 0.00) | .07 | 0.04 (0.02 to 0.06) | < .001 | 0.04 (0.02 to 0.06) | < .001 | 0.01 (-0.00 to 0.03) | .08 | 0.01 (-0.01 to 0.04) | .24 |
| Father’s education | -0.00 (-0.01 to 0.01) | .94 | -0.00 (-0.01 to 0.01) | .94 | 0.00 (0.00 to 0.01) | .01 | 0.00 (0.00 to 0.01) | .01 | 0.00 (0.00 to 0.01) | .04 | 0.00 (-0.00 to 0.01) | .06 |
| Mother’s education | 0.00 (-0.00 to 0.01) | .47 | 0.00 (-0.00 to 0.01) | .46 | 0.00 (0.00 to 0.01) | .02 | 0.00 (0.00 to 0.01) | .02 | 0.00 (-0.00 to 0.00) | .56 | 0.00 (-0.00 to 0.00) | .56 |
| Physician parent | 0.03 (-0.02 to 0.07) | .25 | 0.03 (-0.02 to 0.07) | .27 | 0.03 (0.01 to 0.04) | < .001 | 0.03 (0.01 to 0.04) | < .001 | 0.03 (0.01 to 0.04) | < .001 | 0.03 (0.01 to 0.05) | < .001 |
| High-income families | -0.01 (-0.06 to 0.05) | .83 | -0.01 (-0.08 to 0.06) | .86 | 0.05 (0.03 to 0.07) | < .001 | 0.05 (0.03 to 0.08) | < .001 | 0.07 (0.05 to 0.09) | < .001 | 0.07 (0.05 to 0.10) | < .001 |
| NCEE score | -0.05 (-0.07 to -0.02) | < .001 | -0.05 (-0.08 to -0.01) | .01 | 0.04 (0.03 to 0.05) | < .001 | 0.04 (0.03 to 0.05) | < .001 | 0.01 (0.01 to 0.02) | .001 | 0.01 (-0.02 to 0.04) | .36 |
| Intrinsic motivation | 0.17 (0.14 to 0.19) | < .001 | 0.17 (0.13 to 0.20) | < .001 | 0.09 (0.08 to 0.10) | < .001 | 0.09 (0.08 to 0.10) | < .001 | 0.08 (0.07 to 0.08) | < .001 | 0.08 (0.07 to 0.08) | < .001 |
| Extrinsic motivation | 0.12 (0.10 to 0.14) | < .001 | 0.12 (0.09 to 0.15) | < .001 | 0.07 (0.06 to 0.08) | < .001 | 0.07 (0.06 to 0.08) | < .001 | 0.05 (0.04 to 0.05) | < .001 | 0.05 (0.04 to 0.06) | < .001 |
| **Environmental factors** |  |  |  |  |  |  |  |  |  |  |  |  |
| DFC | -0.10 (-0.16 to -0.03) | .003 | -0.10 (-0.21 to -0.02) | .10 | -0.00 (-0.02 to 0.01) | .63 | -0.00 (-0.04 to 0.03) | .81 | 0.06 (0.04 to 0.08) | < .001 | 0.06 (-0.02 to 0.14) | .14 |
| Long-track programs | -0.10 (-0.19 to -0.01) | .04 | -0.10 (-0.23 to 0.03) | .13 | 0.04 (0.01 to 0.07) | p < 0.01 | 0.04 (0.00 to 0.08) | .05 | 0.18 (0.15 to 0.20) | < .001 | 0.18 (0.09 to 0.26) | < .001 |
| Clinical phase | -0.06 (-0.10 to -0.02) | .002 | -0.06 (-0.16 to 0.04) | .21 | 0.02 (0.01 to 0.03) | p < 0.01 | 0.02 (-0.00 to 0.05) | .11 | -0.34 (-0.35 to -0.33) | < .001 | -0.34 (-0.39 to -0.29) | < .001 |

Abbreviations: DFC = Double First-Class. DFC represents advantaged universities, and non-DFC represents less advantaged universities. NCEE = National College Entrance Examination; Long-track programs = the 5+3 medical education program and 8-year medical education program.

1. 95% confidence intervals are based on robust standard errors clustered at the school level.

# Table S2. Interaction between individual and environmental factor (DFC) in relation to AI literacy

|  | **AI knowledge** | | | | **AI attitude** | | | | **AI behavior** | | | |
| --- | --- | --- | --- | --- | --- | --- | --- | --- | --- | --- | --- | --- |
|  | **Classical SE** | | **Clustered SE ^a^** | | **Classical SE** | | **Clustered SE ^a^** | | **Classical SE** | | **Clustered SE ^a^** | |
|  | **β (95% CI)** | ***p*** | **β (95% CI)** | ***p*** | **β (95% CI)** | ***p*** | **β (95% CI)** | ***p*** | **β (95% CI)** | ***p*** | **β (95% CI)** | ***p*** |
| Male | 0.03 (-0.02 to 0. 07) | .21 | 0.03 (-0.03 to 0. 09) | .38 | 0.04 (0.02 to 0. 05) | < .001 | 0.04 (0.02 to 0. 06) | < .001 | 0.21 (0.19 to 0.22) | < .001 | 0.21 (0.19 to 0.23) | < .001 |
| DFC | -0.16 (-0.42 to 0.09) | .21 | -0.16 (-0.36 to 0.03) | .10 | -0.01 (-0.10 to 0. 07) | .72 | -0.01 (-0.13 to 0. 10) | .81 | 0.07 (-0.01 to 0.15) | .10 | 0.07 (-0.08 to 0.21) | .37 |
| male X DFC | -0.05 (-0.42 to 0.09) | .41 | -0.05 (-0.19 to 0.09) | .46 | 0.07 (0.03 to 0. 10) | .001 | 0.07 (0.01 to 0. 12) | .01 | 0.02 (-0.01 to 0.06) | .20 | 0.02 (-0.02 to 0.07) | .28 |
| Han Chinese | 0.09 (0.03 to 0.16) | .01 | 0.09 (0.02 to 0.16) | .01 | 0.06 (0.04 to 0. 09) | < .001 | 0.06 (0.03 to 0. 10) | < .001 | -0.04 (-0.06 to -0.01) | .001 | -0.04 (-0.09 to -0.02) | .21 |
| Han Chinese X DFC | -0.11 (-0.30 to 0.09) | .27 | -0.11 (-0.24 to 0.02) | .10 | -0.05 (-0.11 to 0. 01) | .13 | -0.05 (-0.13 to 0. 03) | .22 | -0.06 (-0.12 to -0.00) | .05 | -0.06 (-0.16 to -0.04) | .22 |
| Only child | 0.02 (-0.03 to 0.06) | .53 | 0.02 (-0.03 to 0.07) | .53 | -0.01 (-0.03 to 0. 01) | .22 | -0.01 (-0.03 to 0. 01) | .36 | -0.06 (-0.07 to -0.04) | < .001 | -0.06 (-0.09 to -0.03) | .001 |
| Only child X DFC | -0.00 (-0.15 to 0.14) | .95 | -0.00 (-0.14 to 0.13) | .95 | -0.01 (-0.05 to 0. 04) | .80 | -0.01 (-0.05 to 0. 04) | .83 | 0.00 (-0.04 to 0.05) | .89 | 0.00 (-0.05 to 0.06) | .92 |
| Urban | -0.06 (-0.11 to -0.01) | .02 | -0.06 (-0.11 to -0.01) | .02 | 0.04 (0.02 to 0. 06) | < .001 | 0.04 (0.02 to 0. 06) | < .001 | 0.02 (0.00 to 0.04) | .04 | 0.02 (-0.01 to 0.04) | .15 |
| Urban X DFC | 0.18 (0.02 to 0.34) | .03 | 0.18 (-0.01 to 0.36) | .06 | 0.01 (-0.04 to 0. 05) | .81 | 0.01 (-0.04 to 0. 05) | .80 | 0.05 (0.01 to 0.10) | .03 | 0.05 (-0.02 to 0.12) | .14 |
| Father’s education | -0.00 (-0.01 to 0.01) | .86 | -0.00 (-0.01 to 0.01) | .85 | 0.00 (0.00 to 0. 01) | .02 | 0.00 (0.00 to 0. 01) | .01 | 0.00 (0.00 to 0.01) | .03 | 0.00 (0.00 to 0.01) | .03 |
| Father’s education X DFC | 0.00 (-0.02 to 0.02) | .96 | 0.00 (-0.03 to 0.03) | .97 | 0.00 (-0.01 to 0. 01) | .79 | 0.00 (-0.01 to 0. 01) | .80 | 0.00 (-0.01 to 0.01) | .89 | 0.00 (-0.01 to 0.01) | .88 |
| Mother’s education | 0.00 (-0.01 to 0.01) | .68 | 0.00 (-0.01 to 0.01) | .66 | 0.00 (-0.00 to 0. 01) | .06 | 0.00 (-0.00 to 0. 01) | .08 | 0.00 (0.00 to 0.01) | .05 | 0.00 (0.00 to 0.01) | .05 |
| Mother’s education X DFC | 0.01 (-0.01 to 0.03) | .44 | 0.01 (-0.02 to 0.04) | .52 | 0.00 (-0.01 to 0. 01) | .64 | 0.00 (-0.01 to 0. 01) | .59 | 0.00 (-0.00 to 0.01) | .61 | 0.00 (-0.00 to 0.01) | .58 |
| Physician parent | 0.04 (-0.00 to 0.09) | .06 | 0.04 (-0.00 to 0.09) | .06 | 0.03 (0.01 to 0. 05) | < .001 | 0.03 (0.01 to 0. 05) | .001 | 0.05 (0.03 to 0.07) | < .001 | 0.05 (0.03 to 0.07) | < .001 |
| Physician parent X DFC | -0.14 (-0.27 to -0.00) | .05 | -0.14 (-0.27 to -0.00) | .04 | -0.02 (-0.06 to 0. 02) | .33 | -0.02 (-0.06 to 0. 02) | .36 | -0.00 (-0.05 to 0.04) | .81 | -0.00 (-0.04 to 0.03) | .78 |
| High-income families | -0.00 (-0.06 to 0.06) | .96 | -0.00 (-0.08 to 0.07) | .97 | 0.06 (0.04 to 0. 08) | < .001 | 0.06 (0.03 to 0. 08) | < .001 | 0.08 (0.06 to 0.10) | < .001 | 0.08 (0.05 to 0.11) | < .001 |
| High-income families X DFC | -0.02 (-0.18 to 0.15) | .85 | -0.02 (-0.17 to 0.14) | .84 | -0.02 (-0.07 to 0. 03) | .44 | -0.02 (-0.07 to 0. 03) | .45 | -0.03 (-0.07 to 0.02) | .29 | -0.03 (-0.11 to 0.06) | .53 |
| NCEE score | -0.05 (-0.07 to -0.03) | < .001 | -0.05 (-0.09 to -0.01) | .01 | 0.04 (0.03 to 0. 05) | < .001 | 0.04 (0.03 to 0. 05) | < .001 | 0.03 (0.03 to 0.04) | < .001 | 0.03 (0.00 to 0.07) | .04 |
| NCEE score X DFC | 0.01 (-0.05 to 0.07) | .74 | 0.01 (-0.06 to 0.08) | .77 | 0.01 (-0.01 to 0. 03) | .28 | 0.01 (-0.02 to 0. 04) | .45 | -0.02 (-0.04 to 0.00) | .08 | -0.02 (-0.09 to 0.05) | .61 |
| Intrinsic motivation | 0.17 (0.14 to 0.20) | < .001 | 0.17 (0.13 to 0.21) | < .001 | 0.09 (0.08 to 0. 10) | < .001 | 0.09 (0.08 to 0. 10) | < .001 | 0.08 (0.07 to 0.09) | < .001 | 0.08 (0.07 to 0.09) | < .001 |
| Intrinsic motivation X DFC | -0.02 (-0.09 to 0.05) | .62 | -0.02 (-0.11 to 0.07) | .70 | -0.00(-0.02 to 0. 02) | .83 | -0.00(-0.03 to 0. 03) | .87 | -0.02 (-0.05 to -0.00) | .02 | -0.02 (-0.04 to -0.01) | .01 |
| Extrinsic motivation | 0.11 (0.09 to 0.14) | < .001 | 0.11 (0.08 to 0.15) | < .001 | 0.07 (0.06 to 0. 08) | < .001 | 0.07 (0.06 to 0. 08) | < .001 | 0.05 (0.04 to 0.06) | < .001 | 0.05 (0.04 to 0.06) | < .001 |
| Extrinsic motivation X DFC | 0.05 (-0.02 to 0.12) | .17 | 0.05 (-0.02 to 0.12) | .16 | -0.01 (-0.03 to 0. 01) | .38 | -0.01 (-0.03 to 0. 01) | .44 | -0.01 (-0.03 to 0.01) | .41 | -0.01 (-0.03 to 0.01) | .44 |

Abbreviations: DFC = Double First-Class. DFC represents advantaged universities, and non-DFC represents less advantaged universities. NCEE = National College Entrance Examination; Long-track programs = the 5+3 medical education program and 8-year medical education program.

1. 95% confidence intervals are based on robust standard errors clustered at the school level.

# Table S3. The individual and environmental factors associated with AI behavior using different weights

| **DV: AI behavior** | **expert -assigned weights** | | | | **equal weights** | | | |
| --- | --- | --- | --- | --- | --- | --- | --- | --- |
|  | **Classical SE** | | **Clustered SE ^a^** | | **Classical SE** | | **Clustered SE ^a^** | |
|  | **β (95% CI)** | ***p*** | **β (95% CI)** | ***p*** | **β (95% CI)** | ***p*** | **β (95% CI)** | ***p*** |
| Individual factors |  |  |  |  |  |  |  |  |
| Male | 0.20 (0.19 to 0.22) | < .001 | 0.20 (0.18 to 0.22) | < .001 | 0.19 (0.17 to 0.20) | < .001 | 0.19 (0.17 to 0.21) | < .001 |
| Han Chinese | -0.05 (-0.07 to -0.03) | < .001 | -0.05 (-0.10 to 0.01) | .08 | -0.04 (-0.06 to -0.03) | < .001 | -0.04 (-0.10 to 0.01) | .09 |
| Only child | -0.04 (-0.05 to -0.02) | < .001 | -0.04 (-0.06 to -0.01) | .01 | -0.04 (-0.05 to -0.02) | < .001 | -0.04 (-0.06 to -0.01) | .01 |
| Urban | 0.01 (-0.00 to 0.03) | .08 | 0.01 (-0.01 to 0.04) | .24 | 0.02 (0.00 to 0.03) | .04 | 0. 02 (-0.01 to 0.04) | .16 |
| Father’s education | 0.00 (0.00 to 0.01) | .04 | 0.00 (-0.00 to 0.01) | .06 | 0.00 (0.00 to 0.01) | .05 | 0.00 (0.00 to 0.01) | .07 |
| Mother’s education | 0.00 (-0.00 to 0.00) | .56 | 0.00 (-0.00 to 0.00) | .56 | 0.00 (-0.00 to 0.00) | .32 | 0.00 (-0.00 to 0.00) | .31 |
| Physician parent | 0.03 (0.01 to 0.04) | < .001 | 0.03 (0.01 to 0.05) | < .001 | 0.03 (0.02 to 0.05) | < .001 | 0.03 (0.02 to 0.05) | < .001 |
| High-income families | 0.07 (0.05 to 0.09) | < .001 | 0.07 (0.05 to 0.10) | < .001 | 0.07 (0.05 to 0.09) | < .001 | 0.07 (0.05 to 0.10) | < .001 |
| NCEE score | 0.01 (0.01 to 0.02) | .001 | 0.01 (-0.02 to 0.04) | .36 | 0.02 (0.01 to 0.03) | < .001 | 0.02 (-0.01 to 0.05) | .17 |
| Intrinsic motivation | 0.08 (0.07 to 0.08) | < .001 | 0.08 (0.07 to 0.08) | < .001 | 0.08 (0.07 to 0.08) | < .001 | 0.08 (0.07 to 0.08) | < .001 |
| Extrinsic motivation | 0.05 (0.04 to 0.05) | < .001 | 0.05 (0.04 to 0.06) | < .001 | 0.05 (0.04 to 0.06) | < .001 | 0.05 (0.04 to 0.06) | < .001 |
| Environmental factors |  |  |  |  |  |  |  |  |
| DFC | 0.06 (0.04 to 0.08) | < .001 | 0.06 (-0.02 to 0.14) | .14 | 0.06 (0.04 to 0.08) | < .001 | 0.06 (-0.02 to 0.14) | .14 |
| Long-track programs | 0.18 (0.15 to 0.20) | < .001 | 0.18 (0.09 to 0.26) | < .001 | 0.17 (0.14 to 0.19) | < .001 | 0.17 (0.08 to 0.25) | < .001 |
| Clinical phase | -0.34 (-0.35 to -0.33) | < .001 | -0.34 (-0.39 to -0.29) | < .001 | -0.36 (-0.37 to -0.35) | < .001 | -0.36 (-0.41 to -0.31) | < .001 |

Abbreviations: DFC = Double First-Class. DFC represents advantaged universities, and non-DFC represents less advantaged universities. NCEE = National College Entrance Examination; Long-track programs = the 5+3 medical education program and 8-year medical education program.

1. 95% confidence intervals are based on robust standard errors clustered at the school level.

# Table S4. Interaction between individual and environmental factors in relation to AI behavior using different weights

| **DV: AI behavior** | **expert -assigned weights** | | | | **equal weights** | | | |
| --- | --- | --- | --- | --- | --- | --- | --- | --- |
|  | **Classical SE** | | **Clustered SE ^a^** | | **Classical SE** | | **Clustered SE ^a^** | |
|  | **β (95% CI)** | ***p*** | **β (95% CI)** | ***p*** | **β (95% CI)** | ***p*** | **β (95% CI)** | ***p*** |
| Male | 0.21 (0.19 to 0.22) | < .001 | 0.21 (0.19 to 0.23) | < .001 | 0.19 (0.18 to 0.21) | < .001 | 0.19 (0.17 to 0.21) | < .001 |
| DFC | 0.07 (-0.01 to 0.15) | .10 | 0.07 (-0.08 to 0.21) | .37 | 0.08 (-0.01 to 0.16) | .07 | 0.08 (-0.07 to 0.22) | .29 |
| male X DFC | 0.02 (-0.01 to 0.06) | .20 | 0.02 (-0.02 to 0.07) | .28 | 0.03 (-0.01 to 0.06) | .19 | 0.03 (-0.02 to 0.07) | .27 |
| Han Chinese | -0.04 (-0.06 to -0.01) | .001 | -0.04 (-0.09 to 0.02) | .21 | -0.04 (-0.06 to -0.01) | .001 | -0.04 (-0.09 to -0.02) | .24 |
| Han Chinese X DFC | -0.06 (-0.12 to -0.00) | .05 | -0.06 (-0.16 to 0.04) | .22 | -0.06 (-0.12 to 0.00) | .05 | -0.06 (-0.16 to 0.04) | .24 |
| Only child | -0.06 (-0.07 to -0.04) | < .001 | -0.06 (-0.09 to -0.03) | .001 | -0.06 (-0.08 to -0.04) | < .001 | -0.06 (-0.09 to -0.03) | < .001 |
| Only child X DFC | 0.00 (-0.04 to 0.05) | .89 | 0.00 (-0.05 to 0.06) | .92 | -0.01 (-0.05 to 0.04) | .81 | -0.01 (-0.06 to 0.05) | .86 |
| Urban | 0.02 (0.00 to 0.04) | .04 | 0.02 (-0.01 to 0.04) | .15 | 0.02 (0.00 to 0.04) | .02 | 0.02 (-0.00 to 0.05) | .08 |
| Urban X DFC | 0.05 (0.01 to 0.10) | .03 | 0.05 (-0.02 to 0.12) | .14 | 0.05 (0.00 to 0.10) | .03 | 0.05 (-0.02 to 0.12) | .14 |
| Father’s education | 0.00 (0.00 to 0.01) | .03 | 0.00 (0.00 to 0.01) | .03 | 0.00 (0.00 to 0.01) | .03 | 0.00 (0.00 to 0.01) | .03 |
| Father’s education X DFC | 0.00 (-0.01 to 0.01) | .89 | 0.00 (-0.01 to 0.01) | .88 | -0.00 (-0.01 to 0.01) | .91 | -0.00 (-0.01 to 0.01) | .91 |
| Mother’s education | 0.00 (0.00 to 0.01) | .05 | 0.00 (-0.01 to 0.01) | .05 | 0.00 (0.00 to 0.01) | .02 | 0.00 (0.00 to 0.01) | .02 |
| Mother’s education X DFC | 0.00 (-0.00 to 0.01) | .61 | 0.00 (-0.01 to 0.01) | .58 | 0.00 (-0.00 to 0.01) | .57 | 0.00 (-0.00 to 0.01) | .56 |
| Physician parent | 0.05 (0.03 to 0.07) | < .001 | 0.05 (0.03 to 0.07) | < .001 | 0.06 (0.04 to 0.07) | < .001 | 0.06 (0.04 to 0.07) | < .001 |
| Physician parent X DFC | -0.00 (-0.05 to 0.07) | .81 | -0.00 (-0.04 to 0.03) | .78 | -0.01 (-0.05 to 0.03) | .68 | -0.01 (-0.04 to 0.02) | .61 |
| High-income families | 0.08 (0.06 to 0.10) | < .001 | 0.08 (0.05 to 0.11) | < .001 | 0.08 (0.06 to 0.10) | < .001 | 0.08 (0.05 to 0.11) | < .001 |
| High-income families X DFC | -0.03 (-0.07 to 0.02) | .29 | -0.03 (-0.11 to 0.06) | .53 | -0.03 (-0.08 to 0.02) | .27 | -0.03 (-0.11 to 0.06) | .51 |
| NCEE score | 0.03 (0.03 to 0.04) | < .001 | 0.03 (0.00 to 0.07) | .04 | 0.04 (0.03 to 0.05) | < .001 | 0.04 (0.01 to 0.07) | .02 |
| NCEE score X DFC | -0.02 (-0.04 to 0.00) | .08 | -0.02 (-0.09 to 0.05) | .61 | -0.02 (-0.04 to 0.00) | .12 | -0.02 (-0.09 to 0.05) | .65 |
| Intrinsic motivation | 0.08 (0.07 to 0.09) | < .001 | 0.08 (0.07 to 0.09) | < .001 | 0.08 (0.07 to 0.09) | < .001 | 0.08 (0.07 to 0.09) | < .001 |
| Intrinsic motivation X DFC | -0.02 (-0.05 to -0.00) | .02 | -0.02 (-0.04 to -0.01) | .01 | -0.02 (-0.04 to -0.00) | .04 | -0.02 (-0.04 to -0.01) | .01 |
| Extrinsic motivation | 0.05 (0.04 to 0.06) | < .001 | 0.05 (0.04 to 0.06) | < .001 | 0.05 (0.05 to 0.06) | < .001 | 0.05 (0.04 to 0.06) | < .001 |
| Extrinsic motivation X DFC | -0.01 (-0.03 to 0.01) | .41 | -0.01 (-0.03 to 0.01) | .44 | -0.01 (-0.03 to 0.01) | .31 | -0.01 (-0.03 to 0.01) | .33 |

Abbreviations: DFC = Double First-Class. DFC represents advantaged universities, and non-DFC represents less advantaged universities. NCEE = National College Entrance Examination; Long-track programs = the 5+3 medical education program and 8-year medical education program.

1. 95% confidence intervals are based on robust standard errors clustered at the school level.

**References**

1. Zhu J, Li W, Chen L. Doctors in China: improving quality through modernisation of residency education. *Lancet*. 2016;388(10054):1922-1929. doi:10.1016/S0140-6736(16)00582-1
2. Wang W. Medical education in China: progress in the past 70 years and a vision for the future. *BMC Med Educ*. 2021;21(1):453. doi:10.1186/s12909-021-02875-6.
3. Hou J, Michaud C, Li Z, et al. Transformation of the education of health professionals in China: progress and challenges. *Lancet*. 2014;384(9945):819-827. doi:10.1016/S0140-6736(14)61307-6
4. Wu H, Xie A, Yu C, et al. Design and Implementation of the Survey on the Training and Development of Medical Students in China. *Chin J Med Educ*. 2021;41(2):5. doi:10.3760/cma.j.cn115259-20200328-00445. (In Chinese)
5. National Center for Health Professions Education Development (NCHPED). Research Report—The China Medical Student Survey (CMSS) 2021 (Medical Program). Published December, 2021. Accessed January 19, 2025. https://medu.bjmu.edu.cn/cms/show.action?code=publish_4028801e6bb6cf11016be526c0dc0014&siteid=100000&newsid=0b6c0a36692b447384f6b71a39a4863d&channelid=0000000008
6. Qi W. Comment: Programmed to fulfill global ambitions. *Nature*. 2017;545(7655):S53. doi:10.1038/545S53a
7. Wu H, Li S, Zheng J, Guo J. Medical students' motivation and academic performance: the mediating roles of self-efficacy and learning engagement. *Med Educ Online*. 2020;25(1):1742964. doi:10.1080/10872981.2020.1742964
8. Song, J., Chu, Z. & Xu, Y. Policy decoupling in strategic response to the double world-class project: evidence from elite universities in China. *High Educ*. 2021;82: 255–272. doi.org/10.1007/s10734-020-00642-y
